# Supplementary material for: Temporal dynamics of protein complex formation and dissociation during human cytomegalovirus infection
Source: Nat Commun. 2020 Feb 10;11:806. doi: 10.1038/s41467-020-14586-5 (PMC7010728; doi:10.1038/s41467-020-14586-5)
Supplement: Supplementary file 1 — Supplementary Information [file 41467_2020_14586_MOESM1_ESM.pdf]

Temporal dynamics of protein complex formation and dissociation during human cytomegalovirus infection

Hashimoto, et al.

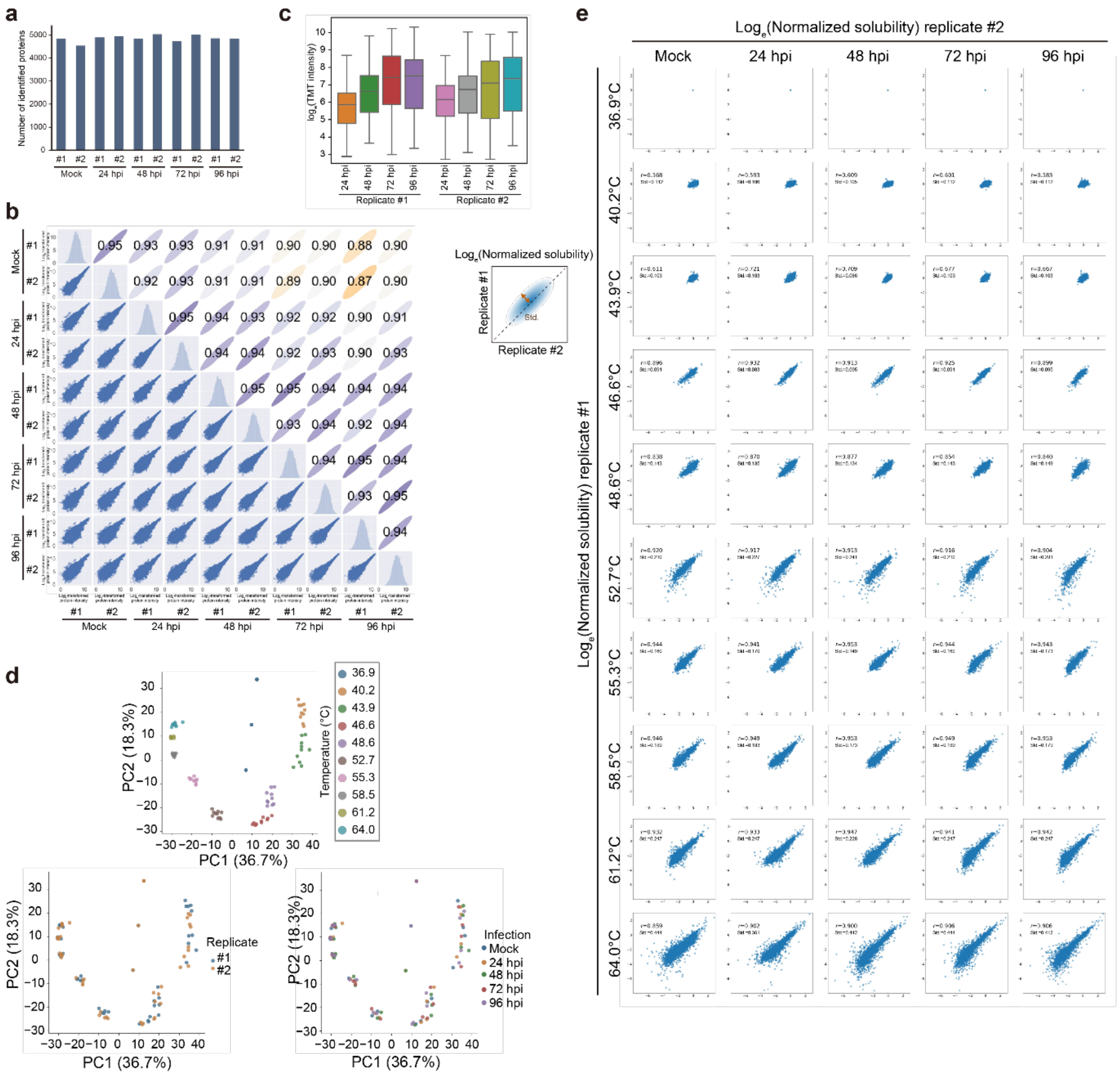

Supplementary Figure 1. Assessment of reproducibility. a Number of identified proteins in each replicate at all time points. **b** Correlation matrix of all proteins between the samples. Correlation coefficients were calculated based on the $\log_e$ transformed abundances, estimated from the sum of the peptide reporter ion intensities for peptides shared across time points for the indicated proteins. Pearson's *r*-values are shown on the top triangle, and the dots for individual protein abundances are plotted on the lower triangle. **c** Comparison of TMT intensities for viral protein abundance detected at different infection time points. Centre line, bounds of box, and

bounds of whiskers represent, respectively, median, quartile, and sample minimum and maximum that are not outliers. **d** PCA plots of all normalized solubilities for temperatures, replicates, and HCMV infection time points. **e** Scatterplots of $\log_e$ transformed normalized adjusted solubility depicting the Pearson correlation coefficient and the standard deviation of the distance to the midline of the data points.

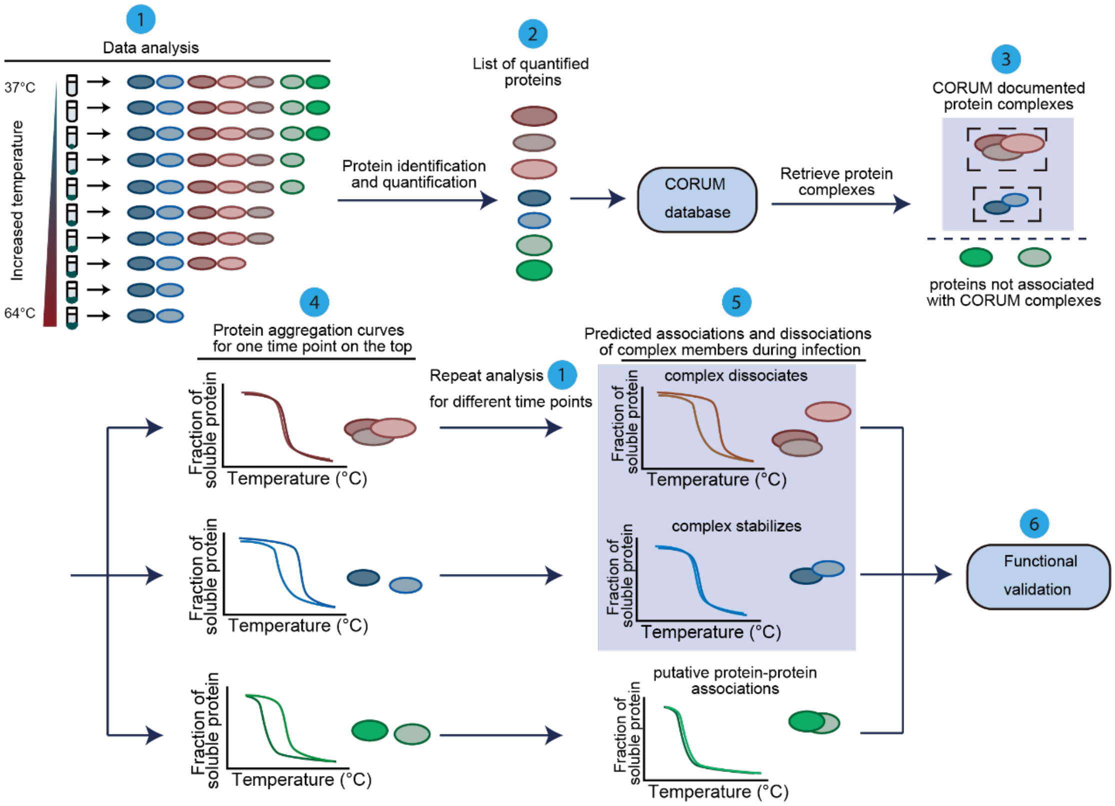

Supplementary Figure 2. Workflow for protein complex analysis. Workflow for analyzing the presence of putative protein complexes and predicting the maintenance, association, and dissociation of members of CORUM protein complexes.

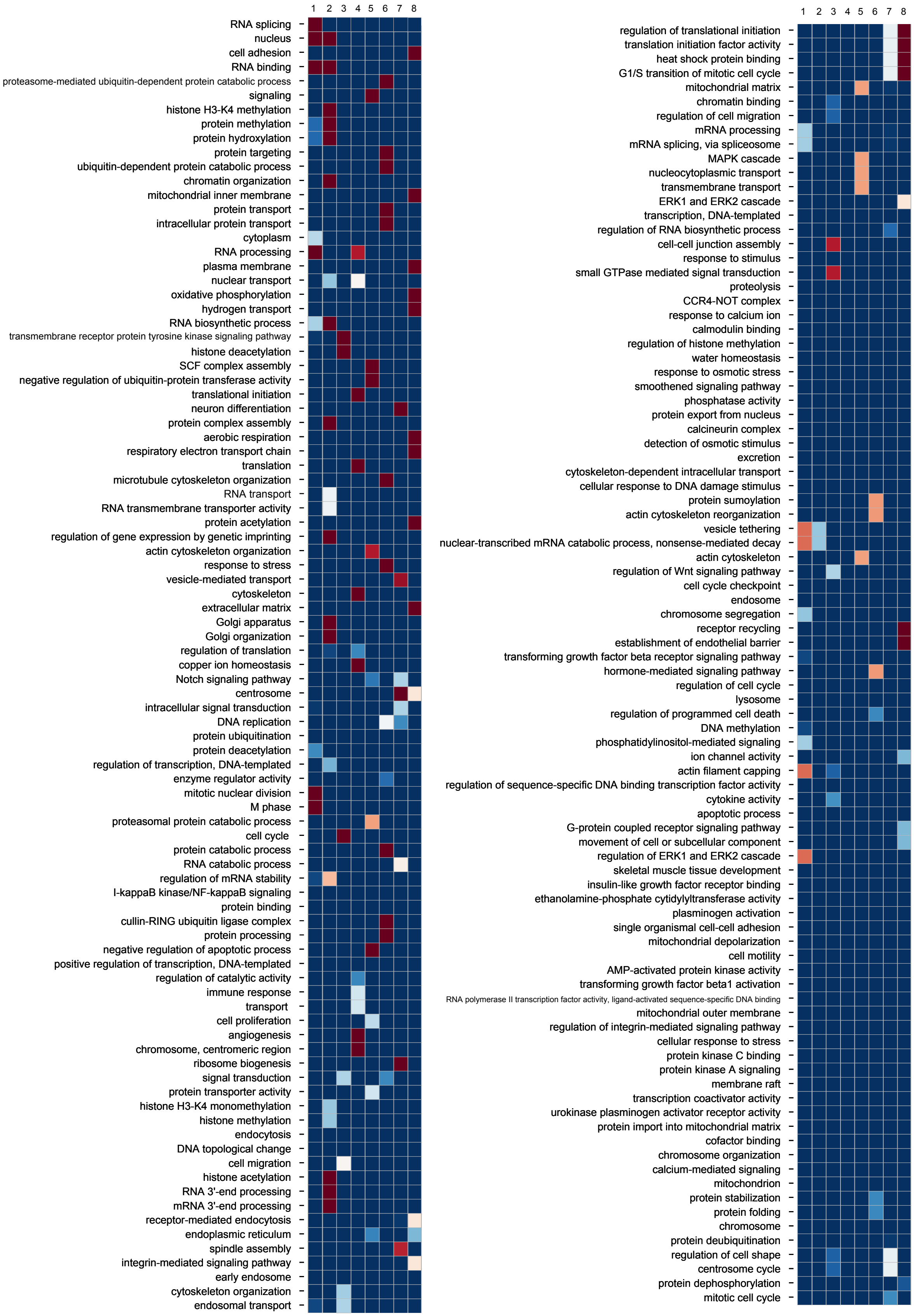
1 2 3 4 5 6 7 8
RNA splicing
nucleus
cell adhesion
RNA binding
proteasome-mediated ubiquitin-dependent protein catabolic process
signaling
histone H3-K4 methylation
protein methylation
protein hydroxylation
protein targeting
ubiquitin-dependent protein catabolic process
chromatin organization
mitochondrial inner membrane
protein transport
intracellular protein transport
cytoplasm
RNA processing
plasma membrane
nuclear transport
oxidative phosphorylation
hydrogen transport
RNA biosynthetic process
transmembrane receptor protein tyrosine kinase signaling pathway
histone deacetylation
SCF complex assembly
negative regulation of ubiquitin-protein transferase activity
translational initiation
neuron differentiation
protein complex assembly
aerobic respiration
respiratory electron transport chain
translation
microtubule cytoskeleton organization
RNA transport
RNA transmembrane transporter activity
protein acetylation
regulation of gene expression by genetic imprinting
actin cytoskeleton organization
response to stress
vesicle-mediated transport
cytoskeleton
extracellular matrix
Golgi apparatus
Golgi organization
regulation of translation
copper ion homeostasis
Notch signaling pathway
centrosome
intracellular signal transduction
DNA replication
protein ubiquitination
protein deacetylation
regulation of transcription, DNA-templated
enzyme regulator activity
mitotic nuclear division
M phase
proteasomal protein catabolic process
cell cycle
protein catabolic process
RNA catabolic process
regulation of mRNA stability
I-kappaB kinase/NF-kappaB signaling
protein binding
cullin-RING ubiquitin ligase complex
protein processing
negative regulation of apoptotic process
positive regulation of transcription, DNA-templated
regulation of catalytic activity
immune response
transport
cell proliferation
angiogenesis
chromosome, centromeric region
ribosome biogenesis
signal transduction
protein transporter activity
histone H3-K4 monomethylation
histone methylation
endocytosis
DNA topological change
cell migration
histone acetylation
RNA 3'-end processing
mRNA 3'-end processing
receptor-mediated endocytosis
endoplasmic reticulum
spindle assembly
integrin-mediated signaling pathway
early endosome
cytoskeleton organization
endosomal transport
1 2 3 4 5 6 7 8
regulation of translational initiation
translation initiation factor activity
heat shock protein binding
G1/S transition of mitotic cell cycle
mitochondrial matrix
chromatin binding
regulation of cell migration
mRNA processing
mRNA splicing, via spliceosome
MAPK cascade
nucleocytoplasmic transport
transmembrane transport
ERK1 and ERK2 cascade
transcription, DNA-templated
regulation of RNA biosynthetic process
cell-cell junction assembly
response to stimulus
small GTPase mediated signal transduction
proteolysis
CCR4-NOT complex
response to calcium ion
calmodulin binding
regulation of histone methylation
water homeostasis
response to osmotic stress
smoothened signaling pathway
phosphatase activity
protein export from nucleus
calcineurin complex
detection of osmotic stimulus
excretion
cytoskeleton-dependent intracellular transport
cellular response to DNA damage stimulus
protein sumoylation
actin cytoskeleton reorganization
vesicle tethering
nuclear-transcribed mRNA catabolic process, nonsense-mediated decay
actin cytoskeleton
regulation of Wnt signaling pathway
cell cycle checkpoint
endosome
chromosome segregation
receptor recycling
establishment of endothelial barrier
transforming growth factor beta receptor signaling pathway
hormone-mediated signaling pathway
regulation of cell cycle
lysosome
regulation of programmed cell death
DNA methylation
phosphatidylinositol-mediated signaling
ion channel activity
actin filament capping
regulation of sequence-specific DNA binding transcription factor activity
cytokine activity
apoptotic process
G-protein coupled receptor signaling pathway
movement of cell or subcellular component
regulation of ERK1 and ERK2 cascade
skeletal muscle tissue development
insulin-like growth factor receptor binding
ethanolamine-phosphate cytidylyltransferase activity
plasminogen activation
single organismal cell-cell adhesion
mitochondrial depolarization
cell motility
AMP-activated protein kinase activity
transforming growth factor beta1 activation
RNA polymerase II transcription factor activity, ligand-activated sequence-specific DNA binding
mitochondrial outer membrane
regulation of integrin-mediated signaling pathway
cellular response to stress
protein kinase C binding
protein kinase A signaling
membrane raft
transcription coactivator activity
urokinase plasminogen activator receptor activity
protein import into mitochondrial matrix
cofactor binding
chromosome organization
calcium-mediated signaling
mitochondrion
protein stabilization
protein folding
chromosome
protein deubiquitination
regulation of cell shape
centrosome cycle
protein dephosphorylation
mitotic cell cycle

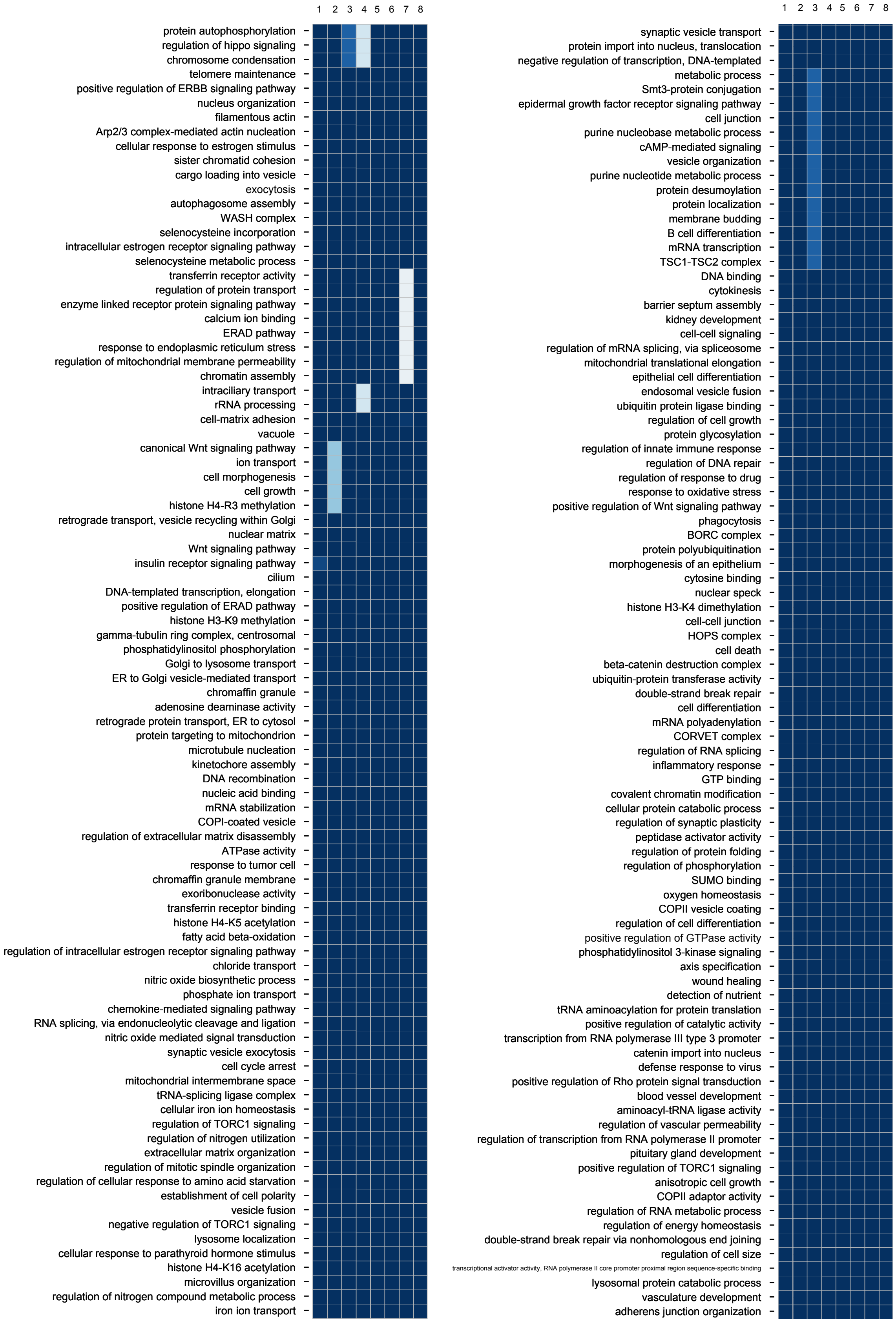
1 2 3 4 5 6 7 8
protein autophosphorylation
regulation of hippo signaling
chromosome condensation
telomere maintenance
positive regulation of ERBB signaling pathway
nucleus organization
filamentous actin
Arp2/3 complex-mediated actin nucleation
cellular response to estrogen stimulus
sister chromatid cohesion
cargo loading into vesicle
exocytosis
autophagosome assembly
WASH complex
selenocysteine incorporation
intracellular estrogen receptor signaling pathway
selenocysteine metabolic process
transferrin receptor activity
regulation of protein transport
enzyme linked receptor protein signaling pathway
calcium ion binding
ERAD pathway
response to endoplasmic reticulum stress
regulation of mitochondrial membrane permeability
chromatin assembly
intraciliary transport
rRNA processing
cell-matrix adhesion
vacuole
canonical Wnt signaling pathway
ion transport
cell morphogenesis
cell growth
histone H4-R3 methylation
retrograde transport, vesicle recycling within Golgi
nuclear matrix
Wnt signaling pathway
insulin receptor signaling pathway
cilium
DNA-templated transcription, elongation
positive regulation of ERAD pathway
histone H3-K9 methylation
gamma-tubulin ring complex, centrosomal
phosphatidylinositol phosphorylation
Golgi to lysosome transport
ER to Golgi vesicle-mediated transport
chromaffin granule
adenosine deaminase activity
retrograde protein transport, ER to cytosol
protein targeting to mitochondrion
microtubule nucleation
kinetochore assembly
DNA recombination
nucleic acid binding
mRNA stabilization
COPI-coated vesicle
regulation of extracellular matrix disassembly
ATPase activity
response to tumor cell
chromaffin granule membrane
exoribonuclease activity
transferrin receptor binding
histone H4-K5 acetylation
fatty acid beta-oxidation
regulation of intracellular estrogen receptor signaling pathway
chloride transport
nitric oxide biosynthetic process
phosphate ion transport
chemokine-mediated signaling pathway
RNA splicing, via endonucleolytic cleavage and ligation
nitric oxide mediated signal transduction
synaptic vesicle exocytosis
cell cycle arrest
mitochondrial intermembrane space
tRNA-splicing ligase complex
cellular iron ion homeostasis
regulation of TORC1 signaling
regulation of nitrogen utilization
extracellular matrix organization
regulation of mitotic spindle organization
regulation of cellular response to amino acid starvation
establishment of cell polarity
vesicle fusion
negative regulation of TORC1 signaling
lysosome localization
cellular response to parathyroid hormone stimulus
histone H4-K16 acetylation
microvillus organization
regulation of nitrogen compound metabolic process
iron ion transport
1 2 3 4 5 6 7 8
synaptic vesicle transport
protein import into nucleus, translocation
negative regulation of transcription, DNA-templated
metabolic process
Smt3-protein conjugation
epidermal growth factor receptor signaling pathway
cell junction
purine nucleobase metabolic process
cAMP-mediated signaling
vesicle organization
purine nucleotide metabolic process
protein desumoylation
protein localization
membrane budding
B cell differentiation
mRNA transcription
TSC1-TSC2 complex
DNA binding
cytokinesis
barrier septum assembly
kidney development
cell-cell signaling
regulation of mRNA splicing, via spliceosome
mitochondrial translational elongation
epithelial cell differentiation
endosomal vesicle fusion
ubiquitin protein ligase binding
regulation of cell growth
protein glycosylation
regulation of innate immune response
regulation of DNA repair
regulation of response to drug
response to oxidative stress
positive regulation of Wnt signaling pathway
phagocytosis
BORC complex
protein polyubiquitination
morphogenesis of an epithelium
cytosine binding
nuclear speck
histone H3-K4 dimethylation
cell-cell junction
HOPS complex
cell death
beta-catenin destruction complex
ubiquitin-protein transferase activity
double-strand break repair
cell differentiation
mRNA polyadenylation
CORVET complex
regulation of RNA splicing
inflammatory response
GTP binding
covalent chromatin modification
cellular protein catabolic process
regulation of synaptic plasticity
peptidase activator activity
regulation of protein folding
regulation of phosphorylation
SUMO binding
oxygen homeostasis
COPII vesicle coating
regulation of cell differentiation
positive regulation of GTPase activity
phosphatidylinositol 3-kinase signaling
axis specification
wound healing
detection of nutrient
tRNA aminoacylation for protein translation
positive regulation of catalytic activity
transcription from RNA polymerase III type 3 promoter
catenin import into nucleus
defense response to virus
positive regulation of Rho protein signal transduction
blood vessel development
aminoacyl-tRNA ligase activity
regulation of vascular permeability
regulation of transcription from RNA polymerase II promoter
pituitary gland development
positive regulation of TORC1 signaling
anisotropic cell growth
COPII adaptor activity
regulation of RNA metabolic process
regulation of energy homeostasis
double-strand break repair via nonhomologous end joining
regulation of cell size
transcriptional activator activity, RNA polymerase II core promoter proximal region sequence-specific binding
lysosomal protein catabolic process
vasculature development
adherens junction organization

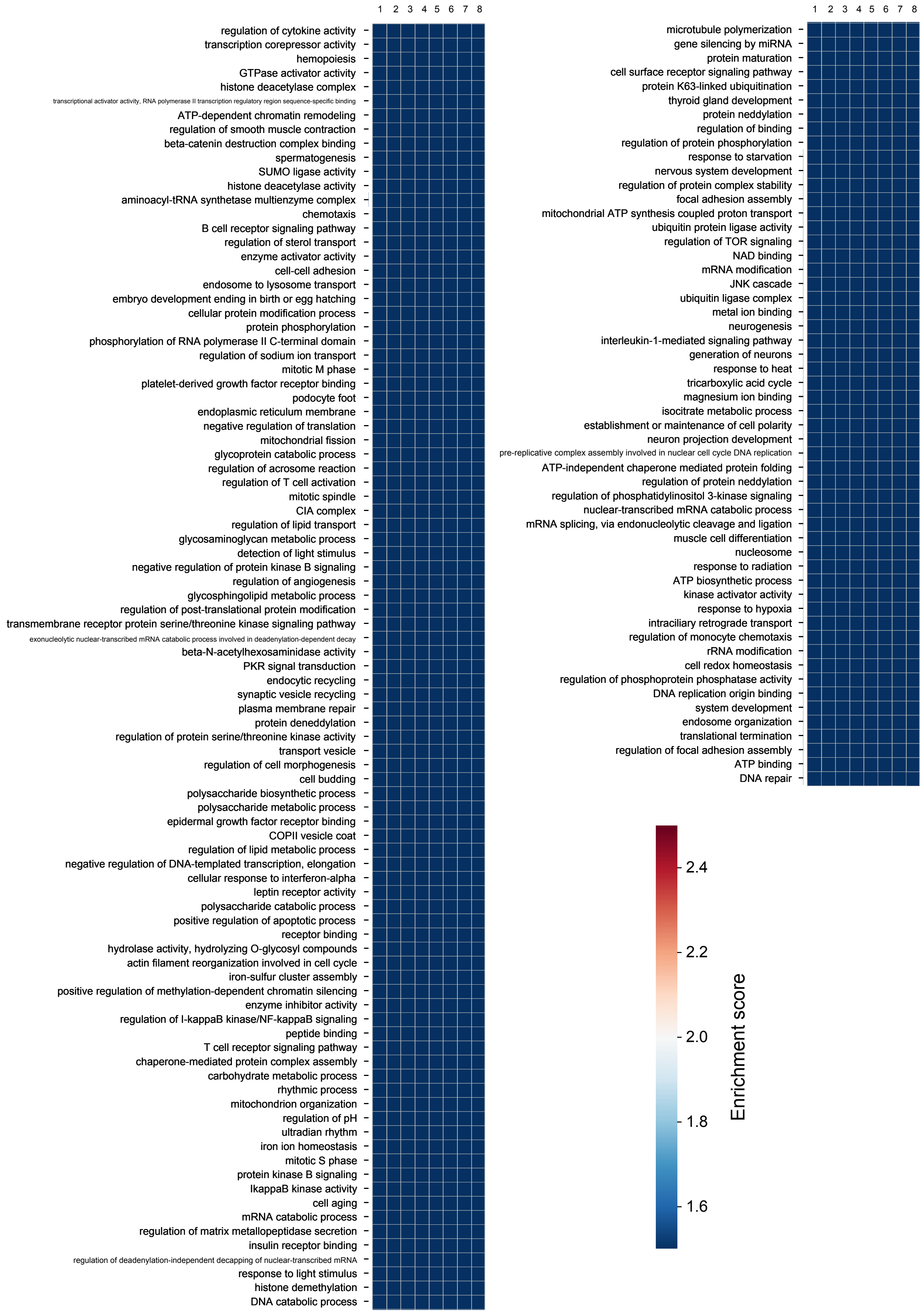
1 2 3 4 5 6 7 8
regulation of cytokine activity
transcription corepressor activity
hemopoiesis
GTPase activator activity
histone deacetylase complex
transcriptional activator activity, RNA polymerase II transcription regulatory region sequence-specific binding
ATP-dependent chromatin remodeling
regulation of smooth muscle contraction
beta-catenin destruction complex binding
spermatogenesis
SUMO ligase activity
histone deacetylase activity
aminoacyl-tRNA synthetase multienzyme complex
chemotaxis
B cell receptor signaling pathway
regulation of sterol transport
enzyme activator activity
cell-cell adhesion
endosome to lysosome transport
embryo development ending in birth or egg hatching
cellular protein modification process
protein phosphorylation
phosphorylation of RNA polymerase II C-terminal domain
regulation of sodium ion transport
mitotic M phase
platelet-derived growth factor receptor binding
podocyte foot
endoplasmic reticulum membrane
negative regulation of translation
mitochondrial fission
glycoprotein catabolic process
regulation of acrosome reaction
regulation of T cell activation
mitotic spindle
CIA complex
regulation of lipid transport
glycosaminoglycan metabolic process
detection of light stimulus
negative regulation of protein kinase B signaling
regulation of angiogenesis
glycosphingolipid metabolic process
regulation of post-translational protein modification
transmembrane receptor protein serine/threonine kinase signaling pathway
exonucleolytic nuclear-transcribed mRNA catabolic process involved in deadenylation-dependent decay
beta-N-acetylhexosaminidase activity
PKR signal transduction
endocytic recycling
synaptic vesicle recycling
plasma membrane repair
protein deneddylation
regulation of protein serine/threonine kinase activity
transport vesicle
regulation of cell morphogenesis
cell budding
polysaccharide biosynthetic process
polysaccharide metabolic process
epidermal growth factor receptor binding
COPII vesicle coat
regulation of lipid metabolic process
negative regulation of DNA-templated transcription, elongation
cellular response to interferon-alpha
leptin receptor activity
polysaccharide catabolic process
positive regulation of apoptotic process
receptor binding
hydrolase activity, hydrolyzing O-glycosyl compounds
actin filament reorganization involved in cell cycle
iron-sulfur cluster assembly
positive regulation of methylation-dependent chromatin silencing
enzyme inhibitor activity
regulation of I-kappaB kinase/NF-kappaB signaling
peptide binding
T cell receptor signaling pathway
chaperone-mediated protein complex assembly
carbohydrate metabolic process
rhythmic process
mitochondrion organization
regulation of pH
ultradian rhythm
iron ion homeostasis
mitotic S phase
protein kinase B signaling
IkappaB kinase activity
cell aging
mRNA catabolic process
regulation of matrix metallopeptidase secretion
insulin receptor binding
regulation of deadenylation-independent decapping of nuclear-transcribed mRNA
response to light stimulus
histone demethylation
DNA catabolic process
1 2 3 4 5 6 7 8
microtubule polymerization
gene silencing by miRNA
protein maturation
cell surface receptor signaling pathway
protein K63-linked ubiquitination
thyroid gland development
protein neddylation
regulation of binding
regulation of protein phosphorylation
response to starvation
nervous system development
regulation of protein complex stability
focal adhesion assembly
mitochondrial ATP synthesis coupled proton transport
ubiquitin protein ligase activity
regulation of TOR signaling
NAD binding
mRNA modification
JNK cascade
ubiquitin ligase complex
metal ion binding
neurogenesis
interleukin-1-mediated signaling pathway
generation of neurons
response to heat
tricarboxylic acid cycle
magnesium ion binding
isocitrate metabolic process
establishment or maintenance of cell polarity
neuron projection development
pre-replicative complex assembly involved in nuclear cell cycle DNA replication
ATP-independent chaperone mediated protein folding
regulation of protein neddylation
regulation of phosphatidylinositol 3-kinase signaling
nuclear-transcribed mRNA catabolic process
mRNA splicing, via endonucleolytic cleavage and ligation
muscle cell differentiation
nucleosome
response to radiation
ATP biosynthetic process
kinase activator activity
response to hypoxia
intraciliary retrograde transport
regulation of monocyte chemotaxis
rRNA modification
cell redox homeostasis
regulation of phosphoprotein phosphatase activity
DNA replication origin binding
system development
endosome organization
translational termination
regulation of focal adhesion assembly
ATP binding
DNA repair
2.4
2.2
2.0
1.8
1.6
Enrichment score

Supplementary Figure 3. GO enrichment analysis highlights protein complexes enriched in distinct temporal profiles (clusters 1-8). a GO enrichment analysis of protein clusters in Fig. 2a. Enriched GO terms without a limit to the number of complexes assigned to a GO term are shown. Number at the top corresponds to the cluster illustrated in Fig. 2a. Color of bins indicates enrichment scores for each GO term during infection. The enrichment scores were based on the p-values of the Fisher's exact test, being calculated as $-\log_{10}$ p-value.

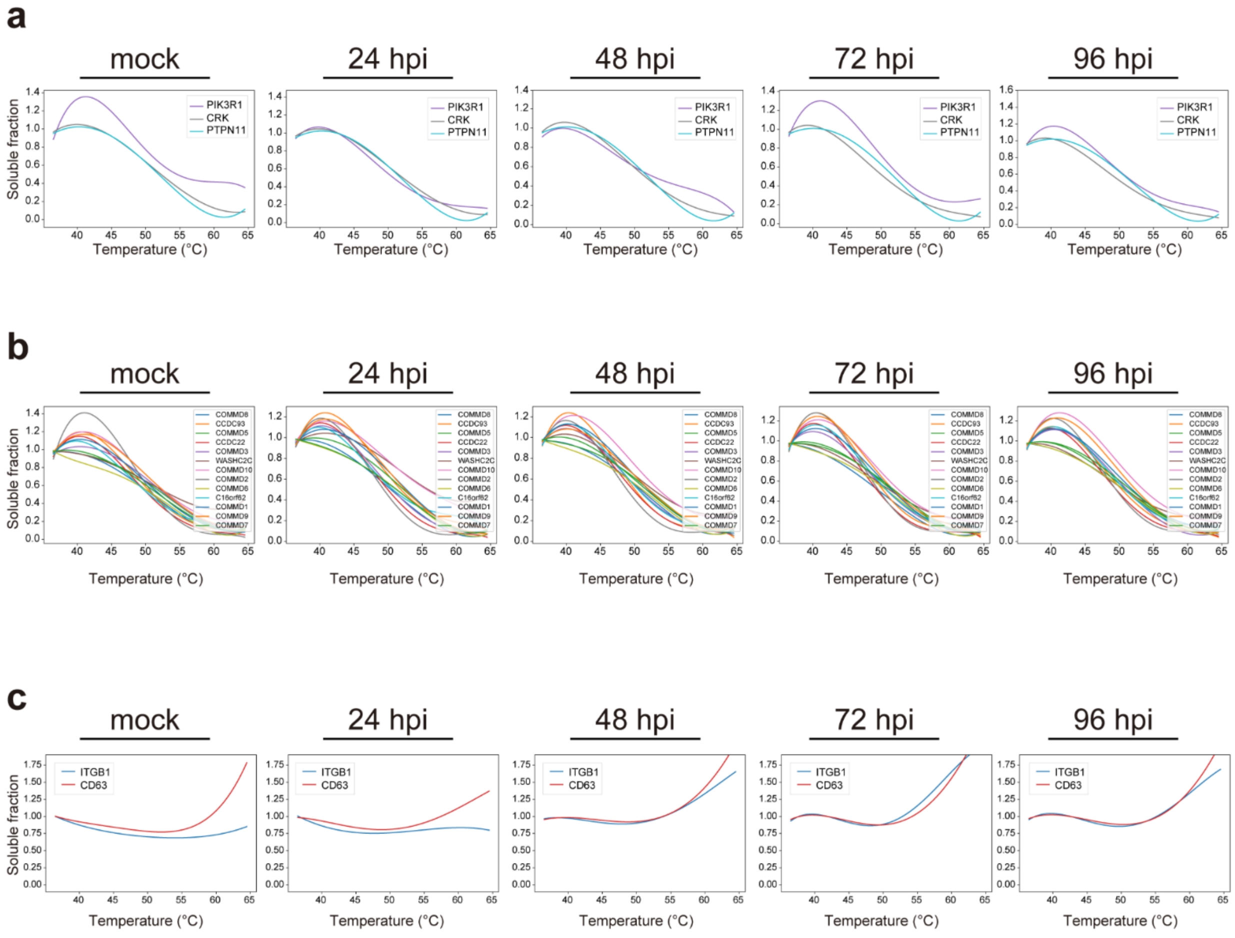

Supplementary Figure 4. Melting curves for selected cellular proteins of interest at different time points of HCMV infection. Melting curves for **a** PI3K pathway proteins, **b** WASHC2C and members of the CCC complex, and **c** ITGB1 and CD63 over the time course of HCMV infection.

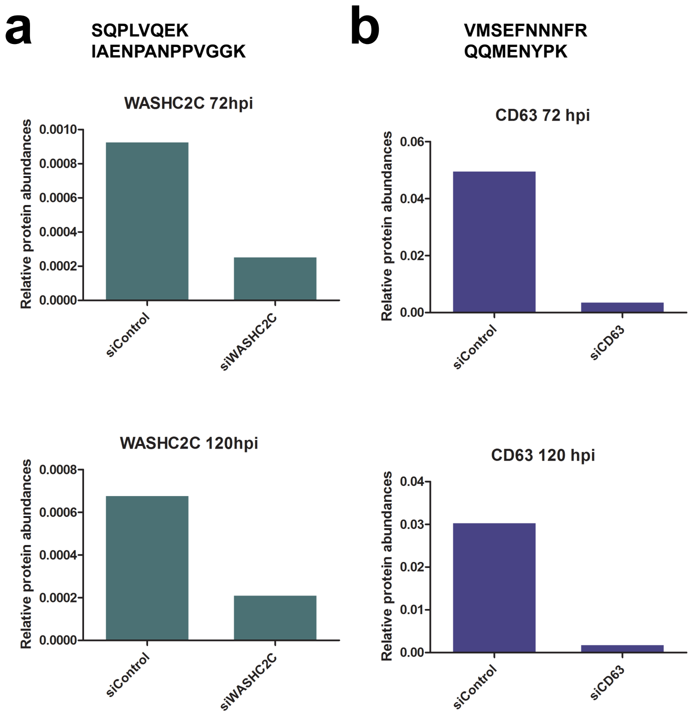

Supplementary Figure 5. siRNA-mediated knockdowns are maintained throughout the HCMV infection. PRM analysis demonstrates that siRNA-mediated knockdowns of **a** WASHC2C and of **b** CD63 are maintained at 72 and 120 hpi. Peptides targeted by PRM analysis for quantification are shown above the plots for each protein.

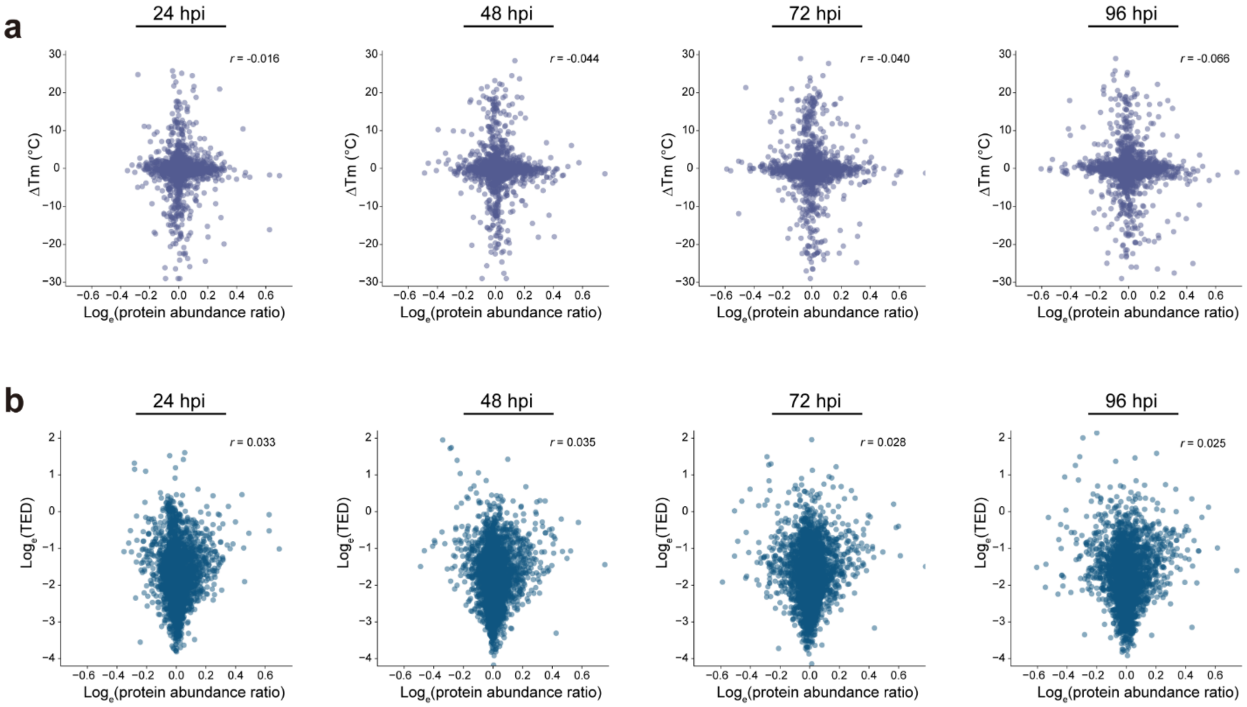

Supplementary Figure 6. Alterations in protein abundance are not the primary driver of changes in ΔTm or TED. a Scatter plot of ΔTm values versus $\log_e$(protein abundance at the indicated time point/protein abundance at mock). Pearson's *r*-value is shown for each infection time point. **b** Scatter plot of $\log_e$(TED) values versus $\log_e$(protein abundance at the indicated time point/protein abundance at mock). Pearson's *r*-value is shown for each infection time point.

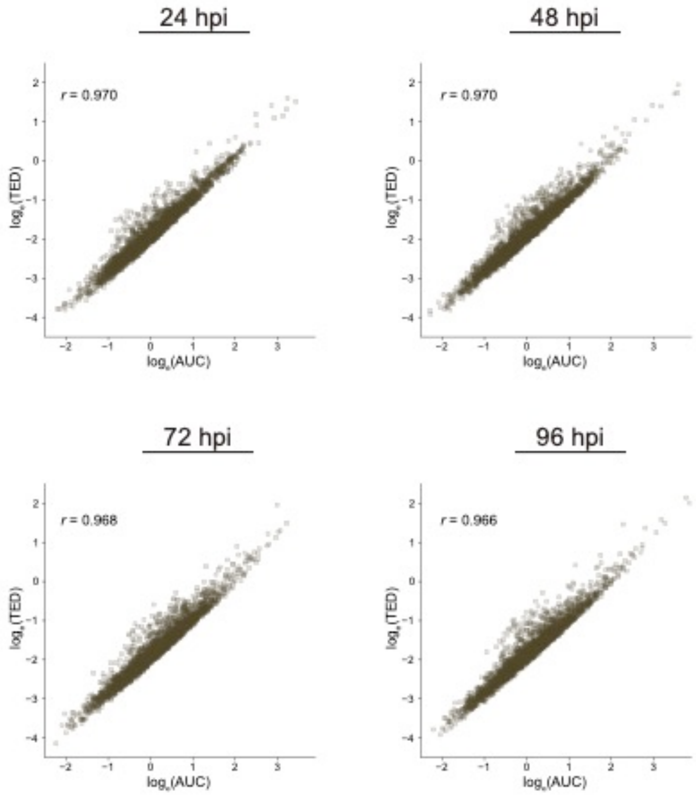

Supplementary Figure 7. Temporal Euclidean distance (TED) correlates with area under the curve (AUC) at every infection time point. Log$_e$(TED) versus log$_e$(AUC) is depicted for each time point during HCMV infection. Pearson's *r*-value is shown for each infection time point.

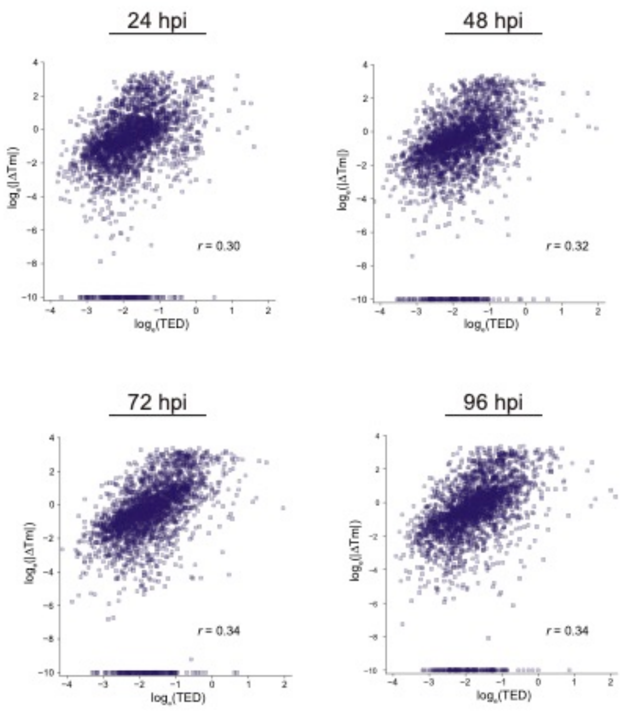

Supplementary Figure 8. TED and ΔTm display positive correlation. Log$_e$(TED) versus log$_e$(|ΔTm|) values are depicted for each time point during HCMV infection. Pearson's *r*-value is shown for each infection time point. Data points that fall at -10 log$_e$|ΔTm| mainly reflect proteins with Tm values greater than 65°C.

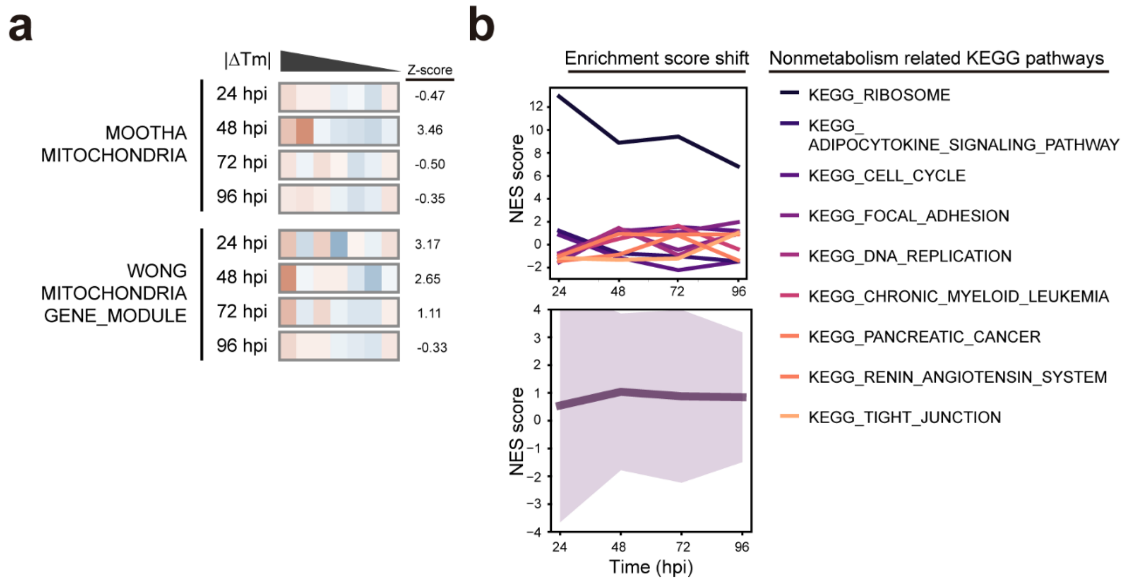

Supplementary Figure 9. Non-mitochondrial gene sets and pathways display varying disruption patterns across infection time. a Enrichment analysis based on |ΔTm| for MOOTHA_MITOCHONDRIA, and WONG_MITOCHONDRIA_GENE_MODULE. Z-scores are shown for each time point. **b** NES score plots for the enriched non-metabolism related pathways. Pathways that display significant changes between early and late stages during infection are selected, among which non-metabolic pathways are plotted. Data represent individual NES scores (top) and the averaged NES scores (bottom) ± SD.

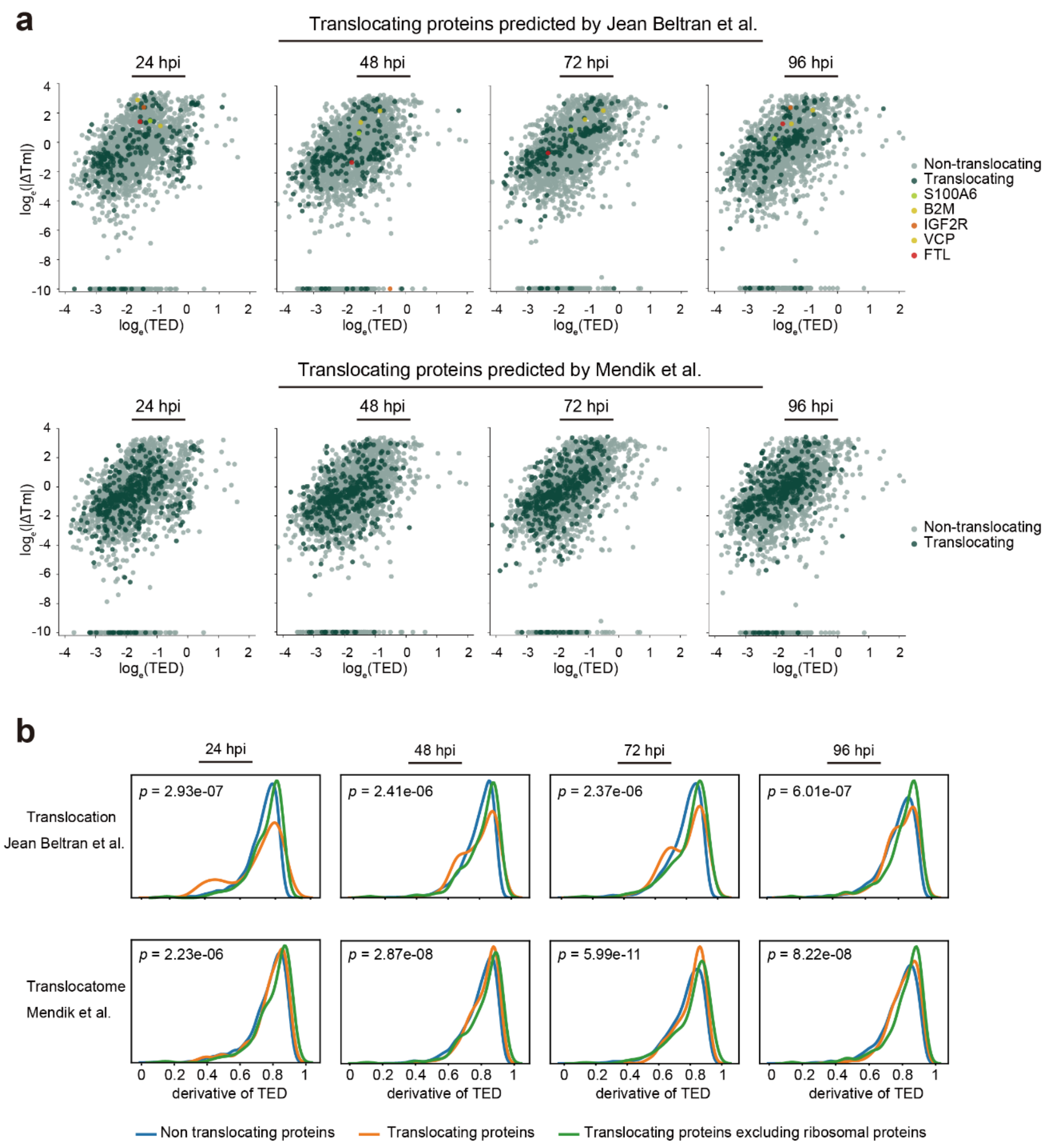

Supplementary Figure 10. TED and Tm values for the majority of translocating proteins are not changed during infection. a Scatter plots of log$_e$(TED) versus log$_e$(|ΔTm|)values at all infection time points are shown, where each dot represents one protein and the dark dots indicate high-confidence translocating proteins documented by the Jean Beltran, et al. (upper) and the translocatome study (lower). Several specific proteins of interest are also indicated. **b**

Distribution of the derivative of TED for non-translocating proteins, all translocating proteins, and translocating proteins excluding ribosomal proteins at each infection time point. Translocating proteins are predicted from Jean Beltran et al. (upper) and the translocatome by Mendik et al. (lower). P-values calculated by a Mann-Whitney U-test on the distribution of TED values between non-translocating proteins and translocating proteins excluding ribosomal proteins are shown.

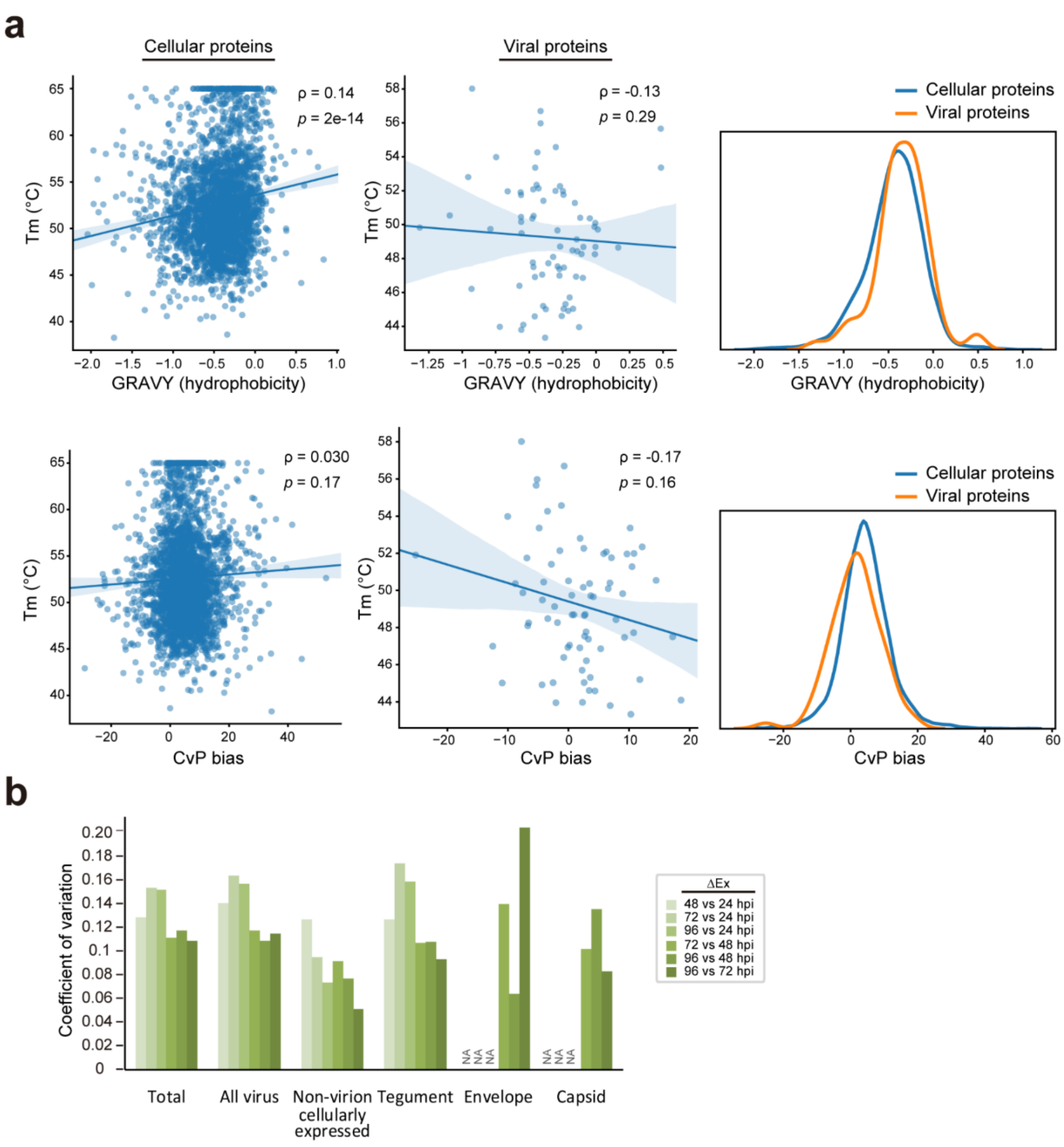

Supplementary Figure 11. Viral proteins have no significant differences from cellular proteins with regard to hydrophobicity or amino acid composition but show variation based on virion compartment. a Top panels: Correlation of average Tm values and GRAVY scores that indicate hydrophobicity for cellular proteins (left) and viral proteins (middle). Spearman's ρ and p values are also shown. The distribution of GRAVY scores of cellular proteins and viral proteins are displayed (right). Bottom panels: Correlation of average Tm values and CvP bias scores for cellular proteins (left) and viral proteins (middle). Spearman's ρ and p values

are also shown. The distribution of CvP bias scores of cellular proteins and viral proteins are displayed (right). **b** Coefficient of variation (CV) of Ex between different infection time points for all proteins, all viral proteins, and viral proteins assigned to specific virion compartments. CVs were not calculated (missing values, NA) for time points with less than 2 proteins.

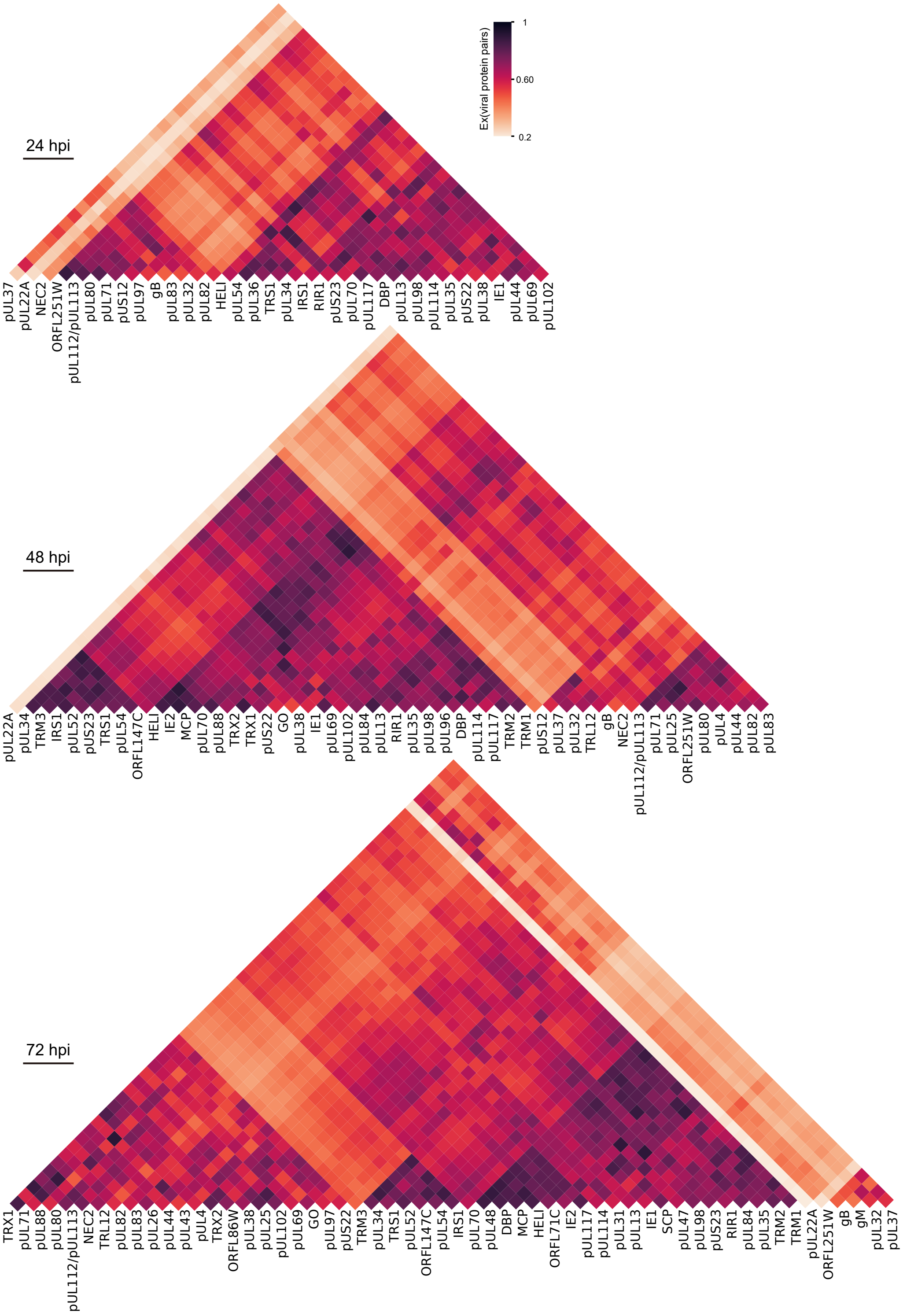

Supplementary Figure 12. Distance matrices delineate potential virus-virus protein associations at 24 hpi, 48 hpi, and 72 hpi. Ex values per pair of viral proteins at 24 hpi (top), 48

hpi (middle), and 72 hpi (bottom). The colors of the bins indicate Ex values for each protein pair at the indicated time point. Viral proteins were clustered by average (UPGMA) method.
