# Supplementary material for: Temporal dynamics of protein complex formation and dissociation during human cytomegalovirus infection
Source: Nat Commun. 2020 Feb 10;11:806. doi: 10.1038/s41467-020-14586-5 (PMC7010728; doi:10.1038/s41467-020-14586-5)
Supplement: Supplementary file 3 — Description of Additional Supplementary Files [file 41467_2020_14586_MOESM3_ESM.docx]

Description of Additional Supplementary Files

Additional descriptions are provided on the first tab of each file.

**Supplementary Data 1. Normalized soluble fraction for aggregation plot**

**Supplementary Data 2. Fuzzy C-means clustering and GO analysis for protein complexes**

**Supplementary Data 3. Enrichment analysis using thermal shift data**

**Supplementary Data 4. Enrichment shift of KEGG pathways during infection**

**Supplementary Data 5. Translocating proteins**

**Supplementary Data 6. Distance matrix of virus proteins**

**Supplementary Data 7. Potential protein complexes, including viral-host interactions, based on COACH algorithm**
