# Supplementary material for: Temporal dynamics of protein complex formation and dissociation during human cytomegalovirus infection
Source: Nat Commun. 2020 Feb 10;11:806. doi: 10.1038/s41467-020-14586-5 (PMC7010728; doi:10.1038/s41467-020-14586-5)
Supplement: Supplementary file 11 — Reporting Summary [file 41467_2020_14586_MOESM11_ESM.pdf]

nature research

Corresponding author(s): Ileana M. Cristea

Last updated by author(s): Jan 6, 2020

Reporting Summary

Nature Research wishes to improve the reproducibility of the work that we publish. This form provides structure for consistency and transparency in reporting. For further information on Nature Research policies, see Authors & Referees and the Editorial Policy Checklist .

Statistics

For all statistical analyses, confirm that the following items are present in the figure legend, table legend, main text, or Methods section.

n/a	Confirmed	
☐	☒	The exact sample size (*n*) for each experimental group/condition, given as a discrete number and unit of measurement
☐	☒	A statement on whether measurements were taken from distinct samples or whether the same sample was measured repeatedly
☐	☒	The statistical test(s) used AND whether they are one- or two-sided<br>*Only common tests should be described solely by name; describe more complex techniques in the Methods section.*
☐	☒	A description of all covariates tested
☐	☒	A description of any assumptions or corrections, such as tests of normality and adjustment for multiple comparisons
☐	☒	A full description of the statistical parameters including central tendency (e.g. means) or other basic estimates (e.g. regression coefficient) AND variation (e.g. standard deviation) or associated estimates of uncertainty (e.g. confidence intervals)
☐	☒	For null hypothesis testing, the test statistic (e.g. *F*, *t*, *r*) with confidence intervals, effect sizes, degrees of freedom and *P* value noted<br>*Give P values as exact values whenever suitable.*
☒	☐	For Bayesian analysis, information on the choice of priors and Markov chain Monte Carlo settings
☒	☐	For hierarchical and complex designs, identification of the appropriate level for tests and full reporting of outcomes
☐	☒	Estimates of effect sizes (e.g. Cohen's *d*, Pearson's *r*), indicating how they were calculated

Our web collection on statistics for biologists contains articles on many of the points above.

Software and code

Policy information about availability of computer code

Data collection	No software was used.
Data analysis	For processing of raw instrument files, MS/MS spectra were analyzed by Proteome Discoverer (PD v2.2, Thermo Fisher Scientific). Additional analyses were conducted using packages in Python (indicated in the respective methods sections). PRM data was analyzed in Skyline (version 4.0). STRING networks were generated in Cytoscape (version 3.7). ImageJ was used for image analysis.

For manuscripts utilizing custom algorithms or software that are central to the research but not yet described in published literature, software must be made available to editors/reviewers. We strongly encourage code deposition in a community repository (e.g. GitHub). See the Nature Research guidelines for submitting code & software for further information.

Data

Policy information about availability of data

All manuscripts must include a data availability statement. This statement should provide the following information, where applicable:

- Accession codes, unique identifiers, or web links for publicly available datasets
- A list of figures that have associated raw data
- A description of any restrictions on data availability

Proteomics data were deposited to the ProteomeXchange Consortium via the PRIDE partner repository with the dataset identifier PXD014747. Any additional data is available upon request.

Field-specific reporting

Please select the one below that is the best fit for your research. If you are not sure, read the appropriate sections before making your selection.

Life sciences study design

All studies must disclose on these points even when the disclosure is negative.

Sample size	A sample size of n = 2 was used for biological replicates for the MS-CETSA analysis and for PRM analyses as these are accepted metrics for these types of experiments. A sample size of n = at least 3 was used for viral titer studies as this is the accepted metric in the field.
Data exclusions	See methods for a detailed description of how data were processed.
Replication	For the proteome studies, the reproducibility in the biological duplicates was supported by the correlation coefficients. All experiments were reliably repeated with at least two biological replicates. The number of replicates for each experiment are indicated in the legend.
Randomization	No group allocation was involved in this study, and all samples were treated identically, so this was not needed.
Blinding	This is not relevant to this study as no groups were allocated and samples were treated identically.

Reporting for specific materials, systems and methods

We require information from authors about some types of materials, experimental systems and methods used in many studies. Here, indicate whether each material, system or method listed is relevant to your study. If you are not sure if a list item applies to your research, read the appropriate section before selecting a response.

Materials & experimental systems

n/a	Involved in the study	
☐	☒	Antibodies
☐	☒	Eukaryotic cell lines
☒	☐	Palaeontology
☒	☐	Animals and other organisms
☒	☐	Human research participants
☒	☐	Clinical data

Methods

n/a	Involved in the study	
☒	☐	ChIP-seq
☒	☐	Flow cytometry
☒	☐	MRI-based neuroimaging

Antibodies

Antibodies used	The primary antibodies that were used in this study are as follows: IE1 primary antibody (clone IB12, Thomas Shenk (Zhu et al., 1995), pUL26 ( ), pUL99 (pp28, clone 10B4, Thomas Shenk (Silva et al., 2003), Integrin B 1 (12G10, Santa Cruz), CD63 (ab118307, abcam), IGF2R (D3V8C, Cell Signaling), ACAT1 (PA5-82154, Thermo Fisher Scientific), and tubulin (T6199 clone DM1A, Sigma). The secondary antibodies that were used in this study are as follows: for IF experiments Alexa 488 and Alexa 555 goat anti-mouse or anti-rabbit IgG (H+L) (Life Technologies) and for WB experiments Alexa 680 and 800 anti-mouse or anti-rabbit IgG (H+L) (Invitrogen).
Validation	The IE1, pUL26, and pUL99 antibodies have been used by multiple publications, and our lab and the group of Thomas Shenk have validated these antibodies in numerous studies. The other commercially available primary and secondary antibodies have been validated by the companies and by several publications.

Eukaryotic cell lines

Policy information about cell lines

Cell line source(s)	Wild type MRC5 human lung fibroblasts and HEK293T cells were purchased from ATCC.
Authentication	No further cell line authentication was conducted.
Mycoplasma contamination	Cells were tested for mycoplasma contamination by ATCC by agar culture, PCR-assay, and Hoechst DNA staining. MRC5 cells were used within ten passages.
Commonly misidentified lines (See ICLAC register)	There are no commonly misidentified lines used.
